# Hierarchical Unilamellar Vesicles of Controlled Compositional Heterogeneity

**DOI:** 10.1371/journal.pone.0050156

**Published:** 2012-11-19

**Authors:** Maik Hadorn, Eva Boenzli, Peter Eggenberger Hotz, Martin M. Hanczyc

**Affiliations:** 1 Department of Physics, Chemistry and Pharmacy, University of Southern Denmark, Odense, Denmark; 2 Department of Informatics, University of Zurich, Zurich, Switzerland; Argonne National Laboratory, United States of America

## Abstract

Eukaryotic life contains hierarchical vesicular architectures (i.e. organelles) that are crucial for material production and trafficking, information storage and access, as well as energy production. In order to perform specific tasks, these compartments differ among each other in their membrane composition and their internal cargo and also differ from the cell membrane and the cytosol. Man-made structures that reproduce this nested architecture not only offer a deeper understanding of the functionalities and evolution of organelle-bearing eukaryotic life but also allow the engineering of novel biomimetic technologies. Here, we show the newly developed vesicle-in-water-in-oil emulsion transfer preparation technique to result in giant unilamellar vesicles internally compartmentalized by unilamellar vesicles of different membrane composition and internal cargo, i.e. hierarchical unilamellar vesicles of controlled compositional heterogeneity. The compartmentalized giant unilamellar vesicles were subsequently isolated by a separation step exploiting the heterogeneity of the membrane composition and the encapsulated cargo. Due to the controlled, efficient, and technically straightforward character of the new preparation technique, this study allows the hierarchical fabrication of compartmentalized giant unilamellar vesicles of controlled compositional heterogeneity and will ease the development of eukaryotic cell mimics that resemble their natural templates as well as the fabrication of novel multi-agent drug delivery systems for combination therapies and complex artificial microreactors.

## Introduction

Giant unilamellar vesicles (GUVs), which represent single aqueous compartments of a diameter of 1 to 100 µm separated from an aqueous surrounding by a single phospholipid bilayer, are intensively studied in different areas of (bio-)chemistry, physics, and in the field of artificial cell synthesis (for a recent review see [1]). Their close analogy to natural cells makes vesicles ideal for the bottom-up analysis of biological processes [2], [3]. Furthermore, their ability to store, transport, and protect distinct chemical cargos, biological and biochemical machineries, and reaction products, enable them to serve as mini-laboratories [4] and as spatially confined bioreactors [5]–[7].

Eukaryotic cells are divided into smaller compartments (e.g. nucleus, vacuoles, mitochondria, endosomes). These highly specialized compartments take over numerous and crucial tasks (e.g. nucleic acid production, material storage, energy production, material degradation) and their evolution is considered as one of the key events in the origin of higher-order life [8]. Internally structured GUVs were proposed not only to achieve a closer resemblance to natural eukaryotic cells, but also for future site-specific multi-agent drug delivery systems [9] with advantageous release characteristics [10] as well as for complex artificial multicompartment microreactors [11]. Consequently, the preparation of compartmentalized vesicles has been investigated for the last three decades. In contrast to multilamellar vesicles (MLVs) which consist of many concentric membranes exhibiting an “onion”-like structure [12], multivesicular vesicles (MVVs) first described as large clusters of smaller compartments sharing common bilayers [13], have been redefined to cover all structures of non-concentric vesicles inside a larger vesicle [14]. Various MVV preparation techniques were reported including the spontaneous [15] or induced [16]–[18] endo-budding of GUVs, the encapsulation of small vesicles by interdigitated lipid sheets [10], [19], the encapsulation of tethered vesicles due to molecular recognition [20], and the formation of double liposomes resulting from the spreading of lipid films on a glass substrate [21], [22] or from reverse phase evaporation [23]. Most of these preparation techniques suffer from intense instrumental manipulation with tedious and multistage procedures. Furthermore, the preparation procedures either lack a control of the lamellarity resulting in biologically implausible and technically limiting multilamellar membranes, or result in compartments of the same membrane composition and/or internal cargo as the confining vesicle. So far, only the rehydration of dried lipid films with an aqueous solution containing small unilamellar vesicles was reported to result in compartmentalized vesicles of heterogeneous composition and of defined lamellarity, i.e. the incorporated non-concentric small unilamellar vesicles (SUV) differed from the confining large unilamellar vesicle (LUV) both by their membrane composition and their internal cargo [11]. However, due to the stochastic character of the incorporation process, the incorporation probability is poor and had to be enhanced by balancing the electrostatic interaction between the encapsulated SUVs and the confining LUV by adjusting the lipid composition of the SUVs and LUVs [24]. Thus, the lack of a separation procedure, the small size both of the compartments (mean diameter: 250 nm) and the compartmentalized vesicles (mean diameter: <2 µm), and the requirement for certain lipid compositions limit the range of applications for the rehydration of dried lipid films.

Here, were report on the vesicle-in-water-in-oil (v/w/o) emulsion transfer with a subsequent separation procedure as a newly developed preparative method for the controlled, efficient, and technically straightforward hierarchical fabrication of giant (i.e. 1–100 µm in diameter) unilamellar vesicles internally compartmentalized by non-concentric giant unilamellar vesicles of different membrane composition and internal cargo. At the core of the novel preparative method is the sequential application of the water-in-oil (w/o) emulsion transfer method reported to result in unilamellar giant vesicles and in a high encapsulation efficiency [25]. Here, for the preparation of the internal compartments, w/o emulsion droplets stabilized by a single layer of phospholipids were forced by centrifugation to pass an interface between a water and oil phase stabilized by another monolayer of phospholipids. During the passage, the two monolayers combined and formed a bilayer that isolated the aqueous lumen of the intermediate GUVs, loaded with sucrose and a fluorophore, from the external aqueous hosting solution containing glucose. For the encapsulation of these intermediate GUVs in larger GUVs, the intermediate GUVs were extruded, transferred to an oil phase, emulsified, and encapsulated within a second GUV population using the same method. GUVs were made of different phospholipid composition as specified below. Thus, the internal compartments were not only loaded with different cargo (i.e. sucrose) but also were comprised of different membrane components (i.e. biotinylated lipids) than the confining GUVs. This method consequently allowed the preparation of what we term as hierarchical unilamellar vesicles (HUVs) with control of the compositional heterogeneity of the involved membranes and cargo to a level, which is not possible with traditional MVV and MLV preparation methods. In addition, our HUV preparation method is characterized by a high yield of HUVs, a high encapsulation efficiency, and a low technical effort.

## Results and Discussion

**Figure 1:**
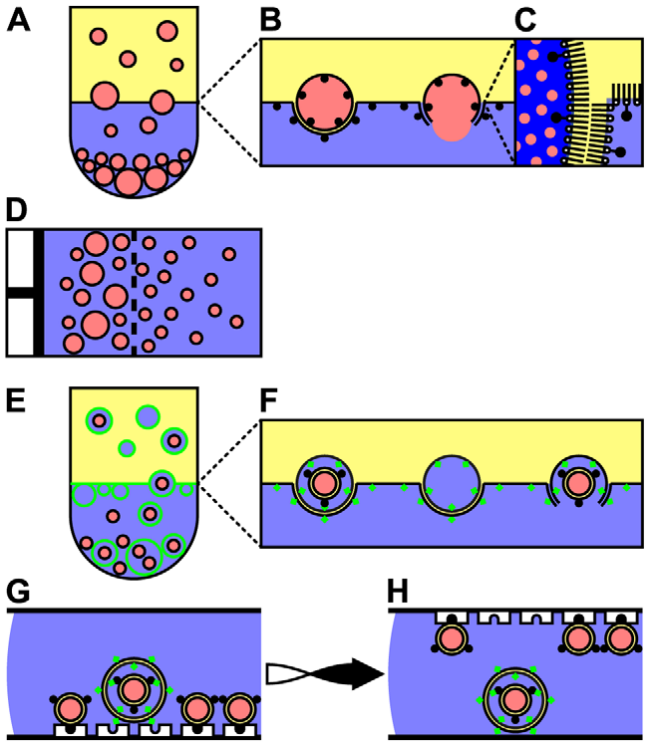
Schematic illustration of the preparation and isolation of intact hierarchical unilamellar vesicles (HUVs). A–C) Preparation of intermediate giant unilamellar vesicles (GUVs) employing the water-in-oil (w/o) emulsion transfer method. Black solid circles indicate biotinylated phospholipids. B, right) Imperfections in either of the two monolayers induce a release of the internal cargo into the hosting solution. C) When passing the water-oil interface the two phospholipid monolayers combine and form a bilayer confining the fluorescently active internal cargo (red circles). D) Repeated extrusion to homogenize the size distribution of the intermediate GUVs. E,F) Preparation of HUVs employing the vesicle-in-water-in-oil emulsion (v/w/o) transfer method. Green solid diamonds indicate fluorescently labeled phospholipids. G, H) Isolation of HUVs from released GUVs using a specially prepared isolation chamber. For details see text.

**Figure 2:**
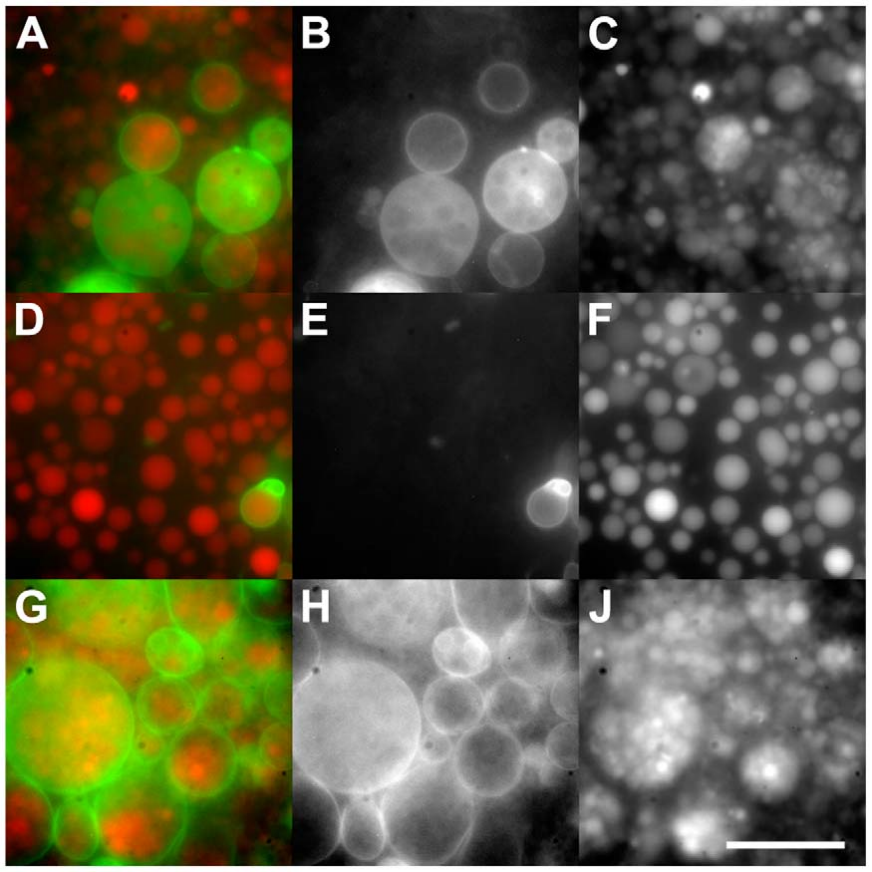
Fluorescence micrographs of intact hierarchical unilamellar vesicles (HUVs) and released giant unilamellar vesicles (GUVs). Intact HUVs and released GUVs before (A–C) and after separation (D–J). A, D, G) Image overlays of the green and red channel micrographs. B, E, H) Separate green channel and C, F, J) separate red channel micrographs. A) The intact HUVs are indicated by the confining membrane fluorescently labeled green and the encapsulated GUVs loaded with a fluorescent cargo (red). In addition, solitary released GUVs not confined by a green labeled membrane are visible. After separation, released GUVs (D) became spatially separated from intact HUVs (G). Scale bar: 25 μm.

Using the w/o emulsion transfer method [25], intermediate GUVs were prepared that encapsulate both sucrose and a fluorescently active marker (Figure 1, red) as internal cargo with a unilamellar bilayer composed of 1% biotinylated and 99% non-biotinylated phospholipids (Figure 1B, left). Although the unilamellarity of the GUVs and the intact HUVs was not analyzed in this study, Pautot et al. [25] provide conclusive experimental data indicating the vesicles prepared by the w/o emulsion transfer method to result in unilamellar giant vesicles, and our microscopic observations corroborate their conclusions. Because the intermediate GUVs containing the disaccharide sucrose were hosted in an external medium that contained the monosaccharide glucose, the intermediate GUVs sedimented and formed a pellet due to their weighted cargo. Vesicles resulting from membrane sheets that resealed after releasing their weighted cargo (cf. Figure 1B, right) had the same density as the surrounding medium and consequently did not sediment and accumulated just below the oil-water interface as reported [26]. These compromised vesicles were removed in the subsequent removal of the supernatant and were therefore not observed when using inverted light and fluorescence microscopy. Extrusion of the sedimented intermediate GUVs led to a vesicle population, which was smaller in radius and less polydisperse (Figure 1D). The vesicle solution (v/w) was then employed as aqueous phase of a subsequent w/o emulsion transfer using fluorescently labeled phospholipids (Figure 1E, green). This v/w/o emulsion transfer resulted in one of three distinct outcomes (Figure 1F): intact HUVs bearing encapsulated GUVs as internal compartments (Figure 1F, left), “empty” HUVs lacking encapsulated GUVs (Figure 1F, middle), and released GUVs originating from intermediate GUVs that were released into the hosting solution due to imperfections in the sealing of the enveloping lipid bilayer (Figure 1F, right). The “empty” HUVs, which lacked the weighted cargo provided by the encapsulated GUVs consequently did not sediment (cf. Figure 1E) and were subsequently removed with the supernatant. The denser disaccharide solution in the encapsulated GUVs acted as weighted cargo in the monosaccharide hosting solution and induced sedimentation of both the intact HUVs and the released GUVs (Figure 2A–C).

To isolate the intact HUVs from the released GUVs, the vesicle solution was transferred to the isolation chamber composed of a streptavidin-coated (Figure 1G, bottom) and a silane-treated cover slip (Figure 1G, top) separated by a spacer. Addition of sodium ions enhanced the binding and reduced dissociation of the streptavidin-biotin linkage [27], and consequently immobilized the released GUVs on the streptavidin coated glass surface. Thus, after turning the isolation chamber upside-down (Figure 1H), the intact HUVs and the released GUVs became vertically separated as the released GUVs remained bound to the streptavidin coated coverslip (Figure 2D–F). The micrographs indicate that this separation technique provides adequate selective enrichment for intact HUVs. The intact HUVs sedimented due to their weighted internal cargo and accumulated in the center of the droplet (Figure 2G–J) due to the evaporation of water along the border of the droplet, which induced a proximal movement of the intact HUVs (cf. moving vesicles in a solute concentration gradient [28]).

**Figure 3:**
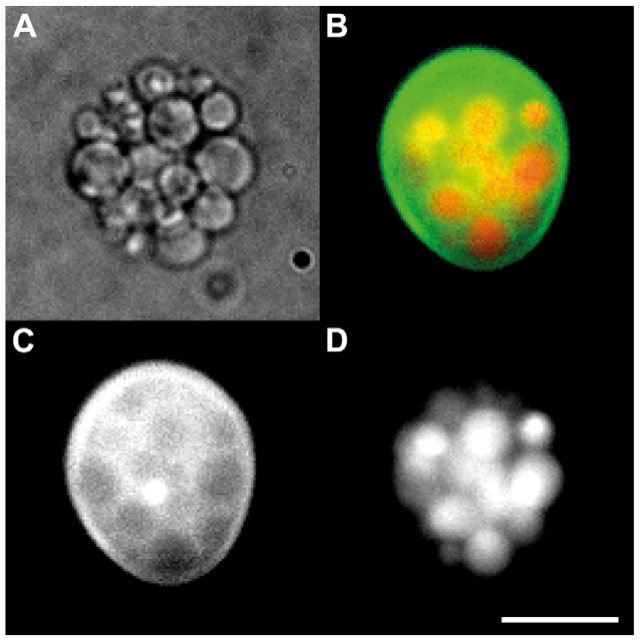
Transmission and fluorescence micrographs of a representative intact hierarchical unilamellar vesicle. A) The unilamellar fluorescently labeled phospholipid membrane confining the densely packed encapsulated giant unilamellar vesicles (GUVs) is only visible in the fluorescence micrographs B and C. B) Image overlay of C) indicating the envelope membrane labeled green and D) indicating the encapsulated GUVs, the lumen of which is labeled red. Scale bar: 10 μm.

Figure 3 shows a representative example of an isolated intact HUV. The encapsulated GUVs are clearly visible in the transmission micrograph (Figure 3A) due to the phase contrast resulting from the differences between the internal disaccharide (i.e. sucrose) and the external monosaccharide (i.e. glucose) medium. The encapsulated GUVs are closely packed inside the confining HUV membrane; the confining HUV membrane is not visible in the transmission micrograph (Figure 3A) due to the lack of a phase contrast resulting from the same internal and external monosaccharide medium. However, the fluorescent HUV membrane confining the encapsulated GUVs is clearly visible in the fluorescence micrographs (Figure 3B, C, green). The fluorescence activity (red) of the cargo of the encapsulated GUVs in Figure 3D shows that the integrity of the encapsulated GUVs remained intact during all manipulations steps (i.e. extrusion, v/w/o emulsion transfer, addition of sodium iodide to the external medium). We speculate the slight deviation from the spherical shape for the intact HUV to originate from the dense packing of the encapsulated GUVs needed to induce sedimentation (see discussion below).

**Figure 4:**
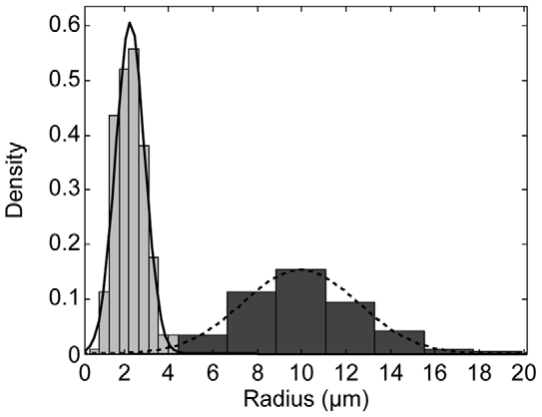
Size distribution of giant unilamellar vesicles (GUVs) and intact hierarchical unilamellar vesicles (HUVs). The size distribution of the GUVs is shown in light gray. The size distribution of the HUVs is shown in dark gray. Lines represent normal fits with mean 2.3 μm and standard deviation 0.7 μm (solid line) and mean 9.9 μm and standard deviation 2.6 μm (dashed line).

Figure 4 shows the size distribution for the released GUVs immobilized on the streptavidin coated glass surface with a mean radius and a standard deviation of 2.27±0.7 µm (Figure 4, light gray) and of the intact HUVs with a mean radius of 9.94±2.6 µm (Figure 4, dark gray). As expected, the size distribution of the GUVs is narrower because of the extrusion.

A reliable counting of the encapsulated GUVs was not available because the small diameter of the GUVs and the resulting spatial rearrangements prevented a proper image analysis (e.g. through confocal laser scanning microscopy). A closer examination of the micrographs (cf. Figure 3) reveals that most of the intact HUVs were densely packed with encapsulated GUVs. It seemed that the larger the diameter of the intact HUVs the more variation of packing density was observable with some of the large intact HUVs only loosely packed with encapsulated GUVs (cf. Figure 2). In order to estimate the minimal number of encapsulated GUVs needed to induce sedimentation within the given time we apply Stokes' law. Because only the cargo of the encapsulated GUVs was of higher density than the surrounding medium, gravitational forces only act on these encapsulated bodies. On the other hand, the buoyancy and drag forces depend on the volume and radius, respectively, of the intact HUV. After reaching the terminal velocity, the gravitational force
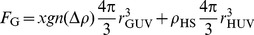
(1)minus the buoyancy force
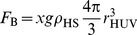
(2)equals the drag force



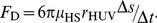
(3)With *x* the rate of centrifugation, *g* = 9.81 m s^−2^ the gravitational acceleration, *n* the number of encapsulated GUVs encapsulated in an intact HUV, *r*_GUV_  = 2.27 µm and *r*_HUV_  = 9.94 µm the radii of the encapsulated GUVs and the intact HUV, Δ*ρ*  =  *ρ*_IS_ – *ρ*_HS_  = 10^−3^ kg m^−3^ the density difference between 15 m% sucrose and 15 m% glucose as the main components of the IS and the HS, µ_HS_  = 1.5956 10^−3^ kg s^−1^ m^−1^ the dynamic viscosity of glucose according to the viscosity prediction of Bui and Nguyen 29], and Δ*s* and Δ*t* the distance to sediment and the time available to complete the sedimentation. We express *n* by relating equations (1), (2), and (3):
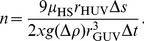
(4)


For the centrifugation values *x*_1_ = 3400, Δ*s*_1_ = 4.2 10^−3^ m, and Δ*t*_1_ = 180 s, the minimal number of encapsulated GUVs needed to induce sedimentation of an intact HUV during the preparation of HUVs is *n*_1_ = 6.2. For the spontaneous sedimentation during separation and imaging with the values *x*_2_ = 1, Δ*s*_2_ = 5 10^−4^ m, and Δ*t*_2_ = 7200 s, the minimal number of encapsulated GUVs needed to induce sedimentation of an intact HUV is *n*_2_ = 63.2. On the other hand, the space available in an intact HUV limits the maximal number of encapsulated GUVs. We assume the encapsulated GUVs to pack like a regular lattice of equal spheres. Consequently, the maximal number of encapsulated GUVs of average size that can be packed in an intact HUV of average size is given by the highest average density of 74% and can be expressed as:
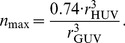
(5)


With the average radii from above *n*_max_ equals 62.1. This indicates that intact HUVs of average size or below have to be packed with encapsulated GUVs as densely as possible in order to sediment for the imaging process. On the other hand, less crowded intact HUVs may have been prepared but did not sediment fast enough to be represented in the micrographs. In order to estimate the smallest radius possible for intact HUVs to sediment during the centrifugation and the spontaneous sedimentation, we express the critical radius of HUVs by relating (4) and (5):
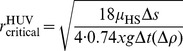
(6)


**Figure 5:**
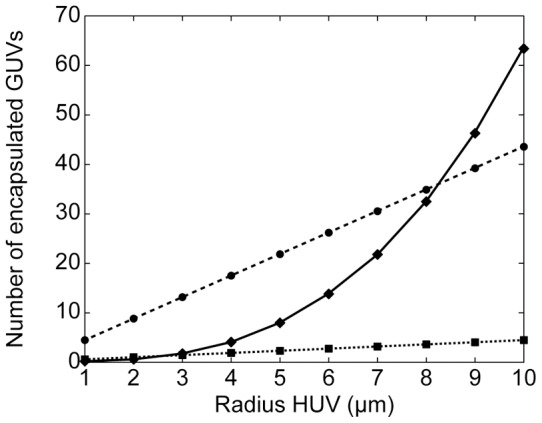
Predictions of the minimal and maximal number of encapsulated giant unilamellar vesicles (GUVs). The minimal number (dashed lines) of encapsulated GUVs needed to induce sedimentation of an intact hierarchical unilamellar vesicle (HUV) of a given size is different for the sedimentation induced by centrifugation (squares) and induced by spontaneous sedimentation (circles). The maximal number (solid line) of encapsulated GUVs that can be packed into an intact HUV of a given size (diamonds) solely depends on the volume available.

Using the values from above reveals 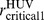  = 2.6 μm for the sedimentation by centrifugation and 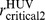  = 8.3 μm for the spontaneous sedimentation during separation and imaging. These values are in good agreement with the size distribution of the intact HUVs (cf. Figure 4) as the majority of intact HUVs represented in the micrographs have a radius larger than 8 µm. Figure 5 details the different regimes defining the formation and architecture of the intact HUVs. For intact HUVs with a radius smaller than 2.6 µm, the minimal number of encapsulated GUVs needed to induce sedimentation exceeds the maximal number of encapsulated GUVs that can be packed into the HUVs. For intact HUVs with a radius between 2.6 µm and 8.3 µm the intact HUVs can be more loosely packed with encapsulated GUVs to induce sedimentation during the preparation process, but even when packed as densely as possible, the number of encapsulated GUVs is insufficient to induce sedimentation during the separation process and consequently these vesicles are not observed during imaging. For intact HUVs with a radius above 8.3 µm the packing can become less dense and these vesicles will still be efficiently sedimented and imaged.

**Figure 6:**
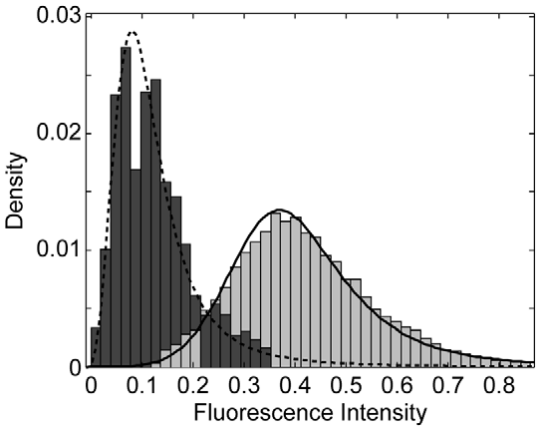
Fluorescence intensity of giant unilamellar vesicles (GUVs) and intact hierarchical unilamellar vesicles (HUVs). The fluorescence intensity of the lumen of immobilized released GUVs is shown in light gray. The fluorescence intensity of the membrane of intact HUVs is shown in dark gray. Lines represent log-logistic fits with mean 0.42 and standard deviation 0.16 (solid line) and mean 0.13 and standard deviation 0.13 (dashed line).

We expect the encapsulation process both of the internal fluorescent cargo (Figure 2, red) and of the fluorescent phospholipids labeling the membranes of the intact HUVs (Figure 2, green) to be stochastic (cf. 30]). Figure 6 shows that the distributions of both signals are well captured by log-logistic distributions and that the variation of the internal cargo distribution (light gray, Figure 6) is larger than the one of the fluorescent membrane marker (dark gray, Figure 6). This is expected, as for a given focal plane the fluorescence signal for the membrane becomes a surface integral whereas the fluorescence signal of the internal cargo becomes a volume integral. Stochastic effects are expected to be larger for a volume than for an area. However, we cannot assess whether this consideration can account for all the differences seen between the GUVs (cf. Figure 2F).

## Conclusions

Preparation of controlled and potentially hierarchical supramolecular structures can be problematic and often requires intense instrumental manipulation. However, the choice of the proper preparation methodology can obviate such issues and result in the custom design and isolation of intact MVVs over incomplete or compromised structures. In this study, we showed that HUVs can be prepared and isolated efficiently by employing the v/w/o emulsion transfer method and by introducing two different selection mechanisms. First, a density difference between disaccharide and monosaccharide solutions allows for positive selectivity for all sucrose-filled and therefore weighted vesicles either solitary, i.e. released GUVs, or encapsulated in HUVs. Second, by controlling different membrane compositions, intact HUVs with the proper outer membrane, i.e. non-biotinylated, were positively selected over the biotinylated released GUVs. The HUV preparation and isolation method proved to be highly efficient, allows control over all encapsulated substances, and ensures the proper composition of HUVs. Future studies may functionalize the membranes of the HUVs (e.g. by the incorporation of membrane proteins) and/or the cargo (e.g. by encapsulation of a biological machinery enabling *in vitro* gene expression) making the preparation of biologically more plausible hierarchical vesicular structures feasible [31]. This will make the HUVs more comparable to multicompartment eukaryotic cells and will ease the fabrication of novel multi-agent drug delivery systems and of complex artificial microreactors.

## Materials and Methods

### Solutions

The intravesicular solution (IS) for the preparation of the intermediate GUVs contained 900 mM sucrose (Sigma-Aldrich, Buchs, Switzerland) and 1 µg/ml Atto 565-Biotin (Sigma-Aldrich, Buchs, Switzerland) as a fluorescent marker. The solution (HS1) to host both the intermediate GUVs and the HUVs contained 900 mM glucose (Sigma-Aldrich, Buchs, Switzerland). For the isolation of intact HUVs, another hosting solution (HS2) was prepared containing 900 mM glucose and 25 mM sodium iodide (Sigma-Aldrich, Buchs, Switzerland). The osmolarity of both hosting solutions (HS1, HS2) was adjusted to match the IS using a vapour pressure osmometer (Vapro5520, ELITech Group, Puteaux, France) while keeping the sodium iodide concentration constant. All solutions were prepared using high quality water (Milli-Q, Millipore, Brussels, Belgium). Prior to use, IS, HS1, and HS2 were filtered using a sterile vacuum-driven Millipore Express Plus Membrane with 0.22 μm pores (Millipore, Brussels, Belgium). The phospholipids were dissolved in light mineral oil (Sigma-Aldrich, Buchs, Switzerland, cat # 330779) to a final concentration of 200 μM and in a molar ratio of 99∶1 of POPC (2-Oleoyl-1-palmitoyl-*sn*-glycero-3-phosphocholine): bPEG2000-DSPE (1,2-Distearoyl-*sn*-Glycero-3-Phosphoethanolamine-N-[Biotinyl(Polyethylene Glycol)2000]) for phospholipid solution 1 (PS1) and of POPC: cfPEG2000-DSPE (1,2-distearoyl-*sn*-glycero- 3-phosphoethanolamine-N-[poly(ethylene glycol)2000-N'-carboxyfluorescein] for PS2. All phospholipids were purchased in chloroform from Avanti Polar Lipids (Alabaster, AL, USA) and used without further purification. Both PSs were prepared as follows: the chloroform was removed from the phospholipid mixture (under vacuum, 60 min), 10 ml of light mineral oil was added and then sonicated for 30 minutes using a Sonorex Digitec DT 156 BH bath sonicator (Bandelin GmbH, Berlin, Germany) thermostated at 50°C, followed by an overnight incubation at room temperature. The PS was protected from light, stored under normal atmosphere at room temperature, and used within one week.

### Preparation of intermediate GUVs

To prepare the intermediate GUVs (cf. Figure 1A), 16 wells of a 96-well microplate with U-shaped bottom (U96 MicroWell plates, polystyrene clear, U-bottom, Thermo Fisher Scientific, Langenselbold, Germany) were filled with 100 μl of HS1, layered with 50 μl of PS1 and incubated for ten minutes. Two 1.5 ml microtubes were filled with 20 μl of IS and 1 ml of PS1. The mixture was mechanically agitated by vigorously grating the microtube over the topside of a 80-well microtube rack (Heathrow Scientific, Nottingham, United Kingdom) to prepare a w/o emulsion. 100 μl of the w/o emulsion were transferred to each of the 16 wells and the microplate was centrifuged for three minutes at 1500 g. Induced by the centrifugation, the droplets passed the oil/water interface. Due to the density difference between HS1 and IS and due to the geometry of the well bottom, the resulting vesicles sedimented and formed a pellet in the center of the well. The PS1 was removed by aspiration using a vacuum pump. Subsequent addition of 200 μl HS1, centrifugation at 1500 g for three minutes, and aspiration by a vacuum pump removed the remaining traces of PS1.

### Extrusion of intermediate GUVs

To homogenize the size distribution of the intermediate GUVs (cf. Figure 1D), the content of all vesicle solutions was pooled and extruded manually using a Mini-Extruder apparatus (Avanti Polar Lipids, Alabaster, AL, USA). The vesicle solution was extruded 21 times through two layers of a polycarbonate filter with a pore size of 12 μm in diameter (Millipore, Brussels, Belgium) at room temperature. The extruded vesicle solution was transferred to three wells of a 96-well microplate with U-bottom and centrifuged (3 minutes, 1500 g). After centrifugation and removal of most of the supernatant, the content of the three wells was transferred to a well of a 96-well microplate with V-shaped bottom (V96 MicroWell plates, polystyrene clear, V-bottom, Thermo Fisher Scientific, Langenselbold, Germany) to ensure tighter accumulation of vesicles after an additional centrifugation (3 minutes, 1500 g).

### Preparation of HUVs

To prepare HUVs (cf. Figure 1E), 10 μl of the extruded intermediate GUVs were transferred to a 1.5 ml microtube containing 200 μl of PS2. The mixture was mechanically agitated as described above to produce a v/w/o emulsion. 150 μl of this v/w/o emulsion were transferred to a well of a 96-well microplate with U-bottom that already contained 100 μl of HS1 layered by 50 μl of PS2 incubated for ten minutes. The intact HUVs (cf. Figure 1F, left) and the released GUVs (cf. Figure 1F, right) both sedimented to the bottom of the well during centrifugation (3 minutes, 3400 g). Subsequent removal of the PS2 by a vacuum pump, addition of 200 μl HS1, centrifugation (3 minutes, 3400 g), and aspiration by a vacuum pump removed the remaining traces of PS2.

### Isolation of intact HUVs

To isolate intact HUVs from released GUVs (cf. Figure 1G, H), an in-house built isolation chamber was used. A streptavidin-coated microscope cover slip (Xenopore Corp., Hawthorne, NJ, USA) was equipped with two layers of tape (label tape, Sigma-Aldrich, Buchs, Switzerland) on the short sides acting as a spacer about 0.5 mm in height. High-vacuum grease (Wacker-Chemie GmbH, Munich, Germany) was then added to the surface of the tape. 10 μl of HS2 and 10 μl of the solution containing the intact HUVs and the released GUVs were successively transferred to the streptavidin-coated microscope cover slip and mixed by repeated aspiration. The isolation chamber was covered with a microscope cover slip previously treated with PlusOne Repel-Silane ES (GE Healthcare, Hillerød, Denmark) according to the supplier's recommendations. For microscopy, the isolation chamber was mounted to a microscope slide. Within two hours both vesicle populations, i.e. intact HUVs and the released GUVs, sedimented to the glass surface (observed by light microscopy). The released GUVs were immobilized due to the interaction of their biotinylated membrane with the bound streptavidin. Multiple turning of the isolation chamber increased the binding probability of biotin and streptavidin: turning the isolation chamber upside-down induced sedimentation of the intact HUVs and unbound released GUVs. After incubation for 60 minutes, the isolation chamber was turned back to its initial state and incubated for 30 minutes. This second incubation with the streptavidin coated surface facing downwards increased the probability of biotinylated released GUVs of being immobilized. After turning the isolation chamber and another incubation for 30 minutes, micrographs were taken from both glass surfaces.

### Microscopy

The vesicles were evaluated in the isolation chamber using an inverted light and fluorescence microscope Nikon Eclipse TE2000-S with a Nikon Intensilight light source, with the following filter settings for the excitation: 455–490 nm (green), 540–585 nm (red), for the dichroic mirror: >495 nm (green), >595 nm (red), and the emission: 499–540 nm (green), 600–650 nm (red). Images were captured with a Photometrics Cascade II 512 camera and in-house software. 10x air, 60x air and 100x oil-immersion objectives (Nikon) were used. The transmission and fluorescence micrographs were automatically (Figure 2) and manually (Figure 3) contrast-adjusted (equally across the entire image) using Adobe Photoshop CS5, version 12.0.4. All figures were prepared using Adobe Illustrator CS5, version 15.0.2.

### Size distribution and fluorescence intensities

The data acquisition for the size distribution (Figure 4) and the fluorescence intensities (Figure 6) of the released GUVs and the intact HUVs was done by manually fitting circles to the margins of visually identified GUVs and HUVs in seven (GUVs) and two (HUVs) micrographs taken with the 100x (GUVs) or the 10x (HUVs) objective. The micrographs showed released GUVs immobilized on a streptavidin-coated cover slip (cf. Figure 2D–F) and intact HUVs after separation (cf. Figure 2G–J). The radius of the circles was adjusted in steps of 0.44 µm (GUVs) and 2.21 µm (HUVs), defining the bin size of the histogram in Figure 4. For Figure 6, the area outside the circles was discarded. From the resulting 9,569,923 (GUVs) and 2,571,451 (HUVs) pixels the upper and lower quartile was removed. From the remaining 9,250,141 and 2,514,398 values 100,000 values were picked randomly for both populations, normalized to 1, and shown in the histograms. Matlab (Matlab R2012a 7.14.0.739, Mathworks, Natwick, MA, USA) was used for randomized sampling, data processing, plotting, and fitting operations.
